# The Translocation and Assembly Module (TAM) of *Edwardsiella tarda* Is Essential for Stress Resistance and Host Infection

**DOI:** 10.3389/fmicb.2020.01743

**Published:** 2020-07-24

**Authors:** Mo-fei Li, Bei-bei Jia, Yuan-yuan Sun, Li Sun

**Affiliations:** ^1^CAS Key Laboratory of Experimental Marine Biology, CAS Center for Ocean Mega-Science, Institute of Oceanology, Chinese Academy of Sciences, Qingdao, China; ^2^Laboratory for Marine Biology and Biotechnology, Pilot National Laboratory for Marine Science and Technology, Qingdao, China; ^3^University of Chinese Academy of Sciences, Beijing, China

**Keywords:** *Edwardsiella tarda*, translocation and assembly module, acid tolerance, membrane integrity, virulence

## Abstract

Translocation and assembly module (TAM) is a protein channel known to mediate the secretion of virulence factors during pathogen infection. *Edwardsiella tarda* is a Gram-negative bacterium that is pathogenic to a wide range of farmed fish and other hosts including humans. In this study, we examined the function of the two components of the TAM, TamA and TamB, of *E. tarda* (named *tamA*_Et_ and *tamB*_Et_, respectively). TamA_Et_ was found to localize on the surface of *E. tarda* and be recognizable by TamA_Et_ antibody. Compared to the wild type, the *tamA* and *tamB* knockouts, TX01Δ*tamA* and TX01Δ*tamB*, respectively, were significantly reduced in motility, flagella formation, invasion into host cells, intracellular replication, dissemination in host tissues, and inducing host mortality. The lost virulence capacities of TX01Δ*tamA* and TX01Δ*tamB* were restored by complementation with the *tamA*_Et_ and *tamB*_Et_ genes, respectively. Furthermore, TX01Δ*tamA* and TX01Δ*tamB* were significantly impaired in the ability to survive under low pH and oxidizing conditions, and were unable to maintain their internal pH balance and cellular structures in acidic environments, which led to increased susceptibility to lysozyme destruction. Taken together, these results indicate that TamA_Et_ and TamB_Et_ are essential for the virulence of *E. tarda* and required for *E. tarda* to survive under stress conditions.

## Introduction

*Edwardsiella tarda* is a Gram-negative bacterium of the family *Enterobacteriaceae*. It is an important pathogen that causes systemic infection in a wide variety of marine and freshwater fish as well as other hosts, including birds, reptiles, and mammals (Mohanty and Sahoo, 2007; Leung et al., 2012, 2019). In aquaculture, *E. tarda*-induced edwardsiellosis in many fish species has led to heavy economic losses in Asia, United States, and Europe (Castro et al., 2006; Nelson et al., 2009; Park et al., 2012). In humans, *E. tarda* has been reported to cause gastroinhastestinal and extraintestinal diseases in immunocompromised people (Abayneh et al., 2013; Shao et al., 2015).

Studies have indicated that *E. tarda* is an intracellular pathogen capable of invading and replicating in host phagocytes and non-phagocytes (Wang et al., 2013; Xie et al., 2014; Sui et al., 2017; Leung et al., 2019). *E. tarda* enters macrophages via both clathrin- and caveolin-mediated endocytosis (Sui et al., 2017). The type III (T3SS) and type VI (T6SS) secretion systems of *E. tarda* inject cytotoxic factors into host cells and facilitate invasion and intracellular replication of the pathogen (Zheng and Leung, 2007; Xie et al., 2010; Akeda et al., 2011). Other virulence factors, such as hemolysins, iron uptake and regulation systems, two-component systems, adhesins, invasins, and lysozyme inhibitors, are also involved in *E. tarda* infections (Wang et al., 2010; Chakraborty et al., 2011; Zheng et al., 2011; Li et al., 2012, 2015; Sun et al., 2012).

Bacterial virulence relies on membrane biogenesis pathways to assemble the outer membrane proteins (OMP) essential for the process of host-pathogen interactions, such as invasion and adhesion (Selkrig et al., 2015). In Gram-negative bacteria, the process of outer membrane assembly is dependent on the translocation and assembly module (TAM) and the β-barrel assembly machinery (BAM) complex (Selkrig et al., 2012; Gruss et al., 2013; Selkrig et al., 2014; Stubenrauch et al., 2016). TAM comprises two subunits: an integral OMP, TamA, and an inner membrane-anchored protein, TamB (Marani et al., 2006; Selkrig et al., 2012). Deletion of *tamA* or *tamB* reduces the virulence of *Klebsiella pneumoniae*, *Proteus mirabilis*, *Citrobacter rodentium*, *Salmonella enterica*, and *Escherichia coli* (Struve et al., 2003; Burall et al., 2004; Kelly et al., 2006; Selkrig et al., 2012). A recent study showed that the assembly of FimD, which is important for the deployment of fimbrial extensions from the surface of bacterial pathogens, and other usher proteins is mediated by the TAM complex (Stubenrauch et al., 2016).

In *E. tarda*, the *tamA* and *tamB* genes have been identified by genome sequencing; however, the role of TamA and TamB in *E. tarda* remains unknown. In the present work, we employed both *in vitro* and *in vivo* approaches to examine the function of *E. tarda tamA* and *tamB* (named *tamA*_Et_ and *tamB*_Et_, respectively). Our results indicated that TamA_Et_ and TamB_Et_ were essential to the infectivity of *E. tarda* and to the survival of *E. tarda* under stress conditions, especially acidic conditions.

## Materials and Methods

### Ethics Statement

The experiments involving live animals in this study were approved by the Ethics Committee of Institute of Oceanology, Chinese Academy of Sciences. All methods were carried out in accordance with the relevant guidelines.

### Fish

Clinically healthy tongue sole (*Cynoglossus semilaevis*) were purchased from a commercial fish farm in Shandong Province, China. Fish were maintained at 20°C in aerated seawater with 144% ± 4% dissolved oxygen (detected with a fast response DO sensor RINKO I; ARO-USB, JFE Advantech Co., Ltd., Japan; Air saturation range: 0–200%). The fish were fed with commercial feed purchased from Shandong Sheng-suo Fish Feed Research Center, Shandong, China. The content (%) of the feed was as follows: protein, ≥45; fat, ≥10; fiber, ≤4; calcium, ≥1.5; phosphate, ≥1.2; lysine, ≥2.2; ashes, ≤17. Before the experiment, the fish were verified to be clinically healthy by examining bacterial presence in spleen, kidney, and liver as reported previously (Zhou and Sun, 2015). Fish were euthanized by immersion in seawater containing 10 mg/L of tricaine methanesulfonate (Sigma, St. Louis, MO, United States) before tissue collection.

### Bacterial Culture

Bacterial strains used in this study are listed in Table 1. *E. tarda* TX01, a pathogenic fish isolate, was cultured in Luria-Bertani broth (LB) at 28°C. The *E. coli* strains were cultured in LB medium at 37°C. Where indicated, polymyxin B, tetracycline, and chloramphenicol were supplemented at the concentrations of 50, 20, and 50 μg/ml, respectively.

**TABLE 1:** Bacterial strains and plasmids used in this study.

Strains or plasmid	Source or reference
*Escherichia coli* strains	
BL21 (DE3)	TransGen Biotech., Beijing, China
DH5α	TransGen Biotech., Beijing, China
S17-1 λ*pir*	Biomedal, Seville, Spain
*Edwardsiella tarda* strains	
TX01	Zhang M. et al., 2008; Cheng et al., 2010
TX01Δ*tamA*	This study
TX01Δ*tamB*	This study
TX01Δ*tamA/tamA*	This study
TX01Δ*tamB/tamB*	This study
Plasmids	
T-A cloning vector T-Simple	TransGen Biotech., Beijing, China
pET259	Zhou and Sun, 2015
pET32a	Novagen, San Diego, CA, United States
pBT3	Zhang W.W. et al., 2008
pJT	Sun et al., 2009
pDM4	Milton et al., 1996
pETTamA	This study
pETTamB	This study
pJTTamA	This study
pJTTamB	This study
pBT3TamA	This study
pBT3TamB	This study
pDMTamA	This study
pDMTamB	This study

### Sequence Analysis

Sequence analysis was performed using the BLAST program at the National Center for Biotechnology Information (NCBI) and the Expert Protein Analysis System. Domain search was performed with the conserved domain search program of NCBI. Theoretical molecular mass and isoelectric point were predicted using EditSeq in the DNASTAR software package (Madison, WI, United States). Multiple sequence alignment was created with DNAMAN. Subcellular localization prediction was performed with the PSORTb v.3.0 server.

### Plasmid Construction

To construct pETTamA, which expresses recombinant TamA_Et_ (rTamA_Et_), *tamA*_Et_ was amplified by PCR with primers TamA-F/TamA-R (Table 2). The PCR products were ligated with the T-A cloning vector T-Simple (TransGen Biotech., Beijing, China), and the recombinant plasmid was restriction digested with *Sma*I to retrieve the *tamA*_Et_-containing fragment, which was inserted into pET259 at the *Swa*I site, resulting in pETTamA. To construct pETTamB, which expresses the TamB domain of TamB_Et_ (amino acid residues 780–1255) with a 6-histidine His-tag at the C-terminus, PCR was conducted with primers TamB-F/TamB-R (Table 2), and the PCR products were inserted into pET259 as above. To construct the low copy-number plasmids pJTTamA and pJTTamB that express *tamA*_Et_ and *tamB_Et_*, respectively, *tamA*_Et_ and *tamB*_Et_ were amplified by PCR with primers TamA-F3/TamA-R and TamB-F3/TamB-R (Table 2), respectively; the PCR products were ligated with the TA cloning vector T-Simple, and the recombinant plasmids were digested with *Sma*I. The fragments containing *tamA*_Et_ and *tamB_Et_* were retrieved and inserted into plasmid pBT3 at the *Eco*RV site, resulting in pBT3TamA and pBT3TamB, respectively. pBT3TamA and pBT3TamB were digested with *Swa*I, and the fragments carrying *tamA*_Et_ and *tamB_Et_* were inserted into plasmid pJT at the *Swa*I site, resulting in pJTTamA and pJTTamB, respectively. All PCR products were verified by sequence analysis.

**TABLE 2:** Primers used in this study.

Primer	Sequence (5′-3′)*^a^*
TamA-F	CCCGGGATGGCTCAGGTCAGATTGGTGGT (*Sma*I)
TamA-R	CCCGGGTAGCTCAGGCCCCAGTCCGA (*Sma*I)
TamA-F3	CCCGGGATGCCACGATTGCGTAGGAT (*Sma*I)
TamB-F	CCCGGGATGCAGCAGGTACAGGGAAATACGC (*Sma*I)
TamB-R	CCCGGGAAACTCAAACTGATAGAGCAGA (*Sma*I)
TamB-F3	CCCGGGATGAGGTGGTATAACATCCG (*Sma*I)
TamA-F1	GGATCCGATAAGGGCGGGGTCAAACA (*Bam*HI)
TamA-R1	CCACCTCAGGCATGCGCCAGCGGCGCCG
TamA-F2	CGCATGCCTGAGGTGGTATAACATCCGG
TamA-R2	GGATCCAGGCGAGTATCCCCTTTCA (*Bam*HI)
TamB-F1	GGATCCCAGACCATCGAGGTGGGGGA (*Bam*HI)
TamB-R1	AGCAGATCCTTGATCACCCCCTGCAGCA
TamB-F2	TGATCAAGGATCTGCTCTATCAGTTTGA
TamB-R2	GGATCCAGAGTACGATCACATTCAGG (*Bam*HI)

**^*a*^*Underlined nucleotides are restriction sites of the enzymes indicated in the parentheses at the end of the sequence.*

### Construction of *tamA*_Et_ and *tamB*_Et_ Knockouts

To construct the mutant *E. tarda* with *tamA*_Et_ knockout, i.e., TX01Δ*tamA*, in-frame deletion of a 1671 bp segment of *tamA*_Et_ (residues 22–578) was performed by overlap extension PCR as follows: the first overlap PCR was performed with primers TamA-F1/TamA-R1, the second overlap PCR was performed with primers TamA-F2/TamA-R2, and the fusion PCR was performed with the primer pair TamA-F1/TamA-R2 (Table 2). The PCR products were ligated into the suicide plasmid pDM4 (Milton et al., 1996) at the *Bgl*II site, resulting in pDMTamA. S17-1 λ*pir* was transformed with pDMTamA, and the transformants were mated with *E. tarda* TX01 via conjugation as previously described (Li et al., 2015). Briefly, the donor strain (resistant to chloramphenicol, sensitive to polymyxin B) and the recipient strain (resistant to polymyxin B, sensitive to chloramphenicol) were cultured in LB medium to OD_600_ of 0.8 and mixed at a ratio of 3:1. The mixture was spread onto a LB agar plate without antibiotics, and the plate was incubated at 28°C for 24 h. After incubation, the bacteria on the plate were resuspended in 2 ml LB, from which 100 μl was taken and plated on a LB agar plate supplemented with polymixin B and chloramphenicol to select for transconjugants. Transconjugants were cured of pDMTamA by incubation on LB agar plates supplemented with 10% sucrose (which induces *sacB*-mediated plasmid curing), and chloramphenicol-sensitive strains were subsequently analyzed by DNA sequencing to confirm in-frame deletion of *tamA*_Et_. This strain was named TX01Δ*tamA*. To construct the mutant *E. tarda* with *tamB*_Et_ knockout, i.e., TX01Δ*tamB*, in-frame deletion of a 987 bp segment of *tamB* (corresponding to amino acid residues 919–1247) was performed by overlap extension PCR as follows: the first overlap PCR was performed with primers TamB-F1/TamB-R1, the second overlap PCR was performed with primers TamB-F2/TamB-R2, and the fusion PCR was performed with the primer pair TamB-F1/TamB-R2 (Table 2). S17-1 λ*pir* was transformed with pDMTamB, and the transconjugants were selected as described above. One of the transconjugants was named TX01Δ*tamB*. To construct the *tamA*_Et_ complement strain TX01Δ*tamA/tamA*, S17-1 λ*pir* was transformed with pJTTamA, and the transformants were conjugated with TX01Δ*tamA*. The transconjugants were selected on LB agar plates supplemented with tetracycline (marker of pJT) and polymyxin B (marker of TX01 and its derivatives). One of the transformants was named TX01Δ*tamA/tamA*. To construct the *tamB*_Et_ complement strain TX01Δ*tamB/tamB*, S17-1 λ*pir* was transformed with pJTTamB, and the transformants were conjugated with TX01Δ*tamB*. The transconjugants were selected as described above. One of the transconjugants was named TX01Δ*tamB/tamB*.

### Purification of Recombinant Proteins and Preparation of Antibodies

*Escherichia coli* BL21 (DE3) was transformed with pETTamA, pETTamB, or pET32a (which expresses the Trx tag). The transformants were cultured in LB medium at 37°C to mid-log phase, and the expression of rTamA_Et_, rTamB_Et_, and rTrx was induced by adding isopropyl-β-D-thiogalactopyranoside to a final concentration of 1 mM. After growth at 16°C for an additional 16 h, the cells were harvested by centrifugation, and recombinant proteins were purified using nickel-nitrilotriacetic acid columns (GE Healthcare, Piscataway, United States) as recommended by the manufacturer. The proteins were treated with Triton X-114 to remove endotoxin as reported previously (Zhang and Sun, 2015). The proteins were dialyzed for 24 h against phosphate buffered saline (PBS) and concentrated using PEG20000 (Solarbio, Beijing, China). The concentrations of the purified proteins were determined using the NanoPhotometer (Implen GmbH, Munich, Germany). Mouse antibodies against rTamA_Et_, rTamB_Et_, and rTrx were prepared as described previously (Li et al., 2016). The antibodies were purified using rProtein G Beads (Solarbio, Beijing, China). The specificity and titer of the antibodies were determined by Western blot and enzyme-linked immunosorbent assay (ELISA) as reported previously (Li et al., 2017).

### Fluorescent Microscopy

Detecting of TamA_Et_ on bacterial surface by fluorescence microscopy was performed as reported previously (Li et al., 2016). Briefly, *E. tarda* TX01 was cultured in LB medium to OD_600_ of 0.8 and resuspended in PBS (pH 7) to 10^8^ CFU/ml. The bacterial suspension was dropped on a glass slide and incubated for 12 h at 28°C. The antibody against rTamA_Et_, rTamB_Et_, or rTrx was added to bacterial suspension. The cells were incubated at 37°C for 1 h and then washed three times with PBS (pH 7). Fluorescein isothiocyanate (FITC)-labeled goat anti-mouse IgG (Abcam, Cambridge, United Kingdom) was added to the bacteria, followed by incubation at 37°C for 1 h in the dark. After staining with 4, 6-diamino-2-phenyl indole (DAPI) (Invitrogen, Carlsbad, CA, United States), bacteria were visualized using a confocal microscope (Carl Zeiss, Oberkochen, Germany). To determine bacterial damage under acidic conditions, *E. tarda* TX01, TX01Δ*tamA*, TX01Δ*tamB*, TX01Δ*tamA/tamA*, and TX01Δ*tamB/tamB* were cultured as above and resuspended in PBS of pH 5 to 10^8^ CFU/ml. The cells were incubated at 28°C for 2 h. After incubation, bacterial cells were treated with propidium iodide (PI) (Majorbio Biotech, Shanghai, China) and DAPI for 15 min in the dark according to the manufacturer’s instructions. The cells were then subjected to microscopy as above.

### Western Blot

*Edwardsiella tarda* TX01, TX01Δ*tamA*, TX01Δ*tamB*, TX01Δ*tamA/tamA*, and TX01Δ*tamB/tamB* were cultured in LB medium to an OD_600_ of 0.8. Whole-cell proteins were prepared and subjected to Western blot as reported previously (Zhang M. et al., 2008) with mouse antibody against rTamB_Et_. RNA polymerase beta was used as an internal reference (Yang et al., 2019) and detected with anti-RNA polymerase beta antibody (Abcam, Cambridge, United Kingdom).

### Electron Microscopy

To examine the flagella of *E. tarda*, *E. tarda* TX01, TX01Δ*tamA*, TX01Δ*tamB*, TX01Δ*tamA/tamA*, and TX01Δ*tamB/tamB* were statically cultured in LB medium at 28°C for 48 h and gently resuspended in PBS. Transmission electron microscope (TEM) examination was performed as previously reported (Givaudan and Lanois, 2000). Briefly, a drop of bacterial suspension was added to carbon-coated copper grids (200 mesh) and rinsed with ultrapure grade water, the bacteria were then negatively stained with 1% (wt/vol) phosphotungstic acid (5 s) and rinsed with ultrapure grade water. The grids were air dried and examined with a TEM (HT7700, Hitachi, Japan). To examine the structure of *E. tarda* under acidic conditions, *E. tarda* TX01, TX01Δ*tamA*, TX01Δ*tamB*, TX01Δ*tamA/tamA*, and TX01Δ*tamB/tamB* were cultured with shaking in LB medium to OD_600_ 0.8, and resuspended in PBS buffer of pH 7 or pH 5 to 10^8^ CFU/ml. The cells were incubated in PBS buffer of different pH at 28°C for 2 h. After incubation, the cells were observed with a TEM as above.

### Motility Assay

*Edwardsiella tarda* TX01, TX01Δ*tamA*, TX01Δ*tamB*, TX01Δ*tamA/tamA*, and TX01Δ*tamB/tamB* were cultured with shaking in LB medium to OD_600_ 0.8, and 10 μl cell suspension were spotted onto the center of fresh LB plates containing 0.3 or 0.5% (w/v) agar. The plates were incubated at 28°C for 2 days, and the motility of the bacteria was assessed by examining the diameter of the bacterial halo on the plate.

### Bacterial Survival Under Acidic and Oxidizing Conditions

PBS buffer was adjusted to pH 7, pH 5, or pH 4.5 with hydrochloric acid. *E. tarda* TX01, TX01Δ*tamA*, TX01Δ*tamB*, TX01Δ*tamA/tamA*, and TX01Δ*tamB/tamB* were cultured as above and resuspended in PBS buffers of different pH to 10^5^ CFU/ml. The cells were incubated at 28°C for 2 h. After incubation, the cells were diluted in PBS (pH 7) and plated on LB agar plates. The plates were incubated at 28°C for 24 h, and the colonies emerged on the plates were counted. The survival rate was calculated as follows: (number of survived cells in different pH/number of survived cells in pH 7) × 100%. To examine the effect of the acidic condition on the internal pH of *E. tarda*, above bacteria were suspended in PBS (pH 5) to 10^10^ CFU/ml and incubated at 28°C for 2 h. The cells were then pelleted by centrifugation and resuspended in 1 ml PBS (pH 7). The cells were boiled for 5 min at 100°C and subjected to sonication in an ice-water bath. The pH of the cell lysate was measured using a pH meter (Sartorius, Beijing, China). To examine the survival of *E. tarda* against lysozyme under acidic condition, TX01, TX01Δ*tamA*, TX01Δ*tamB*, TX01Δ*tamA/tamA* and TX01Δ*tamB/tamB* were suspended in PBS of different pH to 10^5^ CFU/ml and incubated for 2 h. After incubation, the cells were treated with 100 μg/ml hen egg white lysozyme (HEWL) at 28°C for 1 h. The survival rate was calculated as follows: (number of survived cells after lysozyme treatment under different pH/number of survived cells without lysozyme treatment under the corresponding pH) × 100%. To examine the survival of *E. tarda* under oxidizing conditions, above bacteria (10^5^ CFU/ml) were incubated in PBS (pH 7) containing 1 mM, 2 mM, or 3 mM H_2_O_2_ at 28°C for 2 h. The survival rate was calculated as follows: (number of survived cells after H_2_O_2_ treatment/number of cells surviving without H_2_O_2_ treatment) × 100%.

### *In vitro* Infection

#### Bacterial Infection of Peripheral Blood Leukocytes (PBL)

Blood was collected from the caudal vein of tongue sole. PBL were isolated from the blood with 61% Percoll and collected as described previously (Li et al., 2017). The cells were cultured in L-15 medium (Thermo Scientific HyClone, Beijing, China) in 96-well culture plates (10^5^ cells/well). *E. tarda* TX01, TX01Δ*tamA*, TX01Δ*tamB*, TX01Δ*tamA/tamA*, and TX01Δ*tamB/tamB* were prepared as above and added to PBL (10^5^ CFU/well). The cells were incubated at 28°C for 0.5, 1, or 2 h. After incubation, the plates were washed with PBS (pH 7), and the cells were lysed with 100 μl PBS containing 1% Triton X-100. The cell lysate was diluted and plated in triplicate on LB agar plates. The plates were incubated at 28°C for 48 h, and the colonies that emerged on the plates were counted. The genetic identities of the colonies were verified by PCR with specific primers and sequence analysis of the PCR products. The experiment was performed three times.

#### Intracellular

*Edwardsiella tarda* TX01, TX01Δ*tamA*, TX01Δ*tamB*, TX01 Δ*tamA/tamA*, and TX01Δ*tamB/tamB* were prepared as above and added to tongue sole PBL (10^5^ CFU/well). The cells were incubated at 28°C for 1 h and washed three times with PBS (pH 7). Fresh L-15 medium containing 100 μg/ml gentamicin (Solarbio, Beijing, China) was added to the cells, and the cells were incubated at 28°C for 1 h to kill extracellular bacteria. The plates were washed three times with PBS (pH 7) and incubated at 28°C for 0, 1, 2, 4, and 8 h. After incubation, the cells were lysed, and bacterial recovery was determined as above. The experiment was performed three times.

### *In vivo* Infection

*In vivo* infection was performed as reported previously (Li et al., 2015). Briefly, *E. tarda* TX01, TX01Δ*tamA*, TX01Δ*tamB*, TX01Δ*tamA/tamA*, and TX01Δ*tamB/tamB* were cultured as above. The cells were washed with PBS (pH 7) and resuspended in PBS (pH 7) to 5 × 10^6^CFU/ml. Tongue sole (average 15.7 g) were randomly divided into five groups (15 fish/group) and infected via intramuscular injection with 100 μl TX01, TX01Δ*tamA*, TX01Δ*tamB*, TX01Δ*tamA/tamA* or TX01Δ*tamB/tamB*. At 12, 24, and 48 h post-infection, kidney, spleen, and blood were collected from the fish (five at each time point). The tissues were homogenized in PBS (pH 7). The homogenates was serially diluted and plated in triplicate on LB agar plates. The plates were incubated at 28°C for 48 h, and the colonies that appeared on the plates were enumerated. The genetic identity of the colonies was verified as above. For mortality analysis, five groups (20 fish/group) of tongue sole were infected as above with TX01, TX01Δ*tamA*, TX01Δ*tamB*, TX01Δ*tamA/tamA*, or TX01Δ*tamB/tamB*, and the fish were monitored daily for mortality for 15 days.

### Statistical Analysis

All experiments were performed three times. Statistical analyses were carried out with SPSS 17.0 software (SPSS Inc., Chicago, IL, United States). Data were analyzed with analysis of variance (ANOVA), and statistical significance was defined as *P* < 0.05.

## Results

### Characterization of the Sequences of TamA_Et_ and TamB_Et_

A search of the genome of *E. tarda* revealed the presence of *tamA* and *tamB* homologs (named *tamA*_Et_ and *tamB*_Et_, respectively). TamA_Et_ is composed of 578 amino acid residues, with a predicted molecular mass of 64.27 kDa and a predicted pI of 9.1. TamB_Et_ is composed of 1255 amino acid residues, with a predicted molecular mass of 134.99 kDa and a predicted pI of 9.3. TamA_Et_ possesses an outer membrane translocation and assembly module A domain; TamB_Et_ possesses an outer membrane translocation and assembly module B domain (Supplementary Figure S1). TamA_Et_ has 67.1–97.8% overall sequence identities with other bacterial OMP assembly factors, but only 12.8% sequence identity with *E. tarda* BamA. TamB_Et_ has 59.8–96.4% overall sequence identities with other bacterial TamB (Supplementary Figure S2).

### Localization of TamA_Et_ Onto Bacterial Surface

**FIGURE 1:**
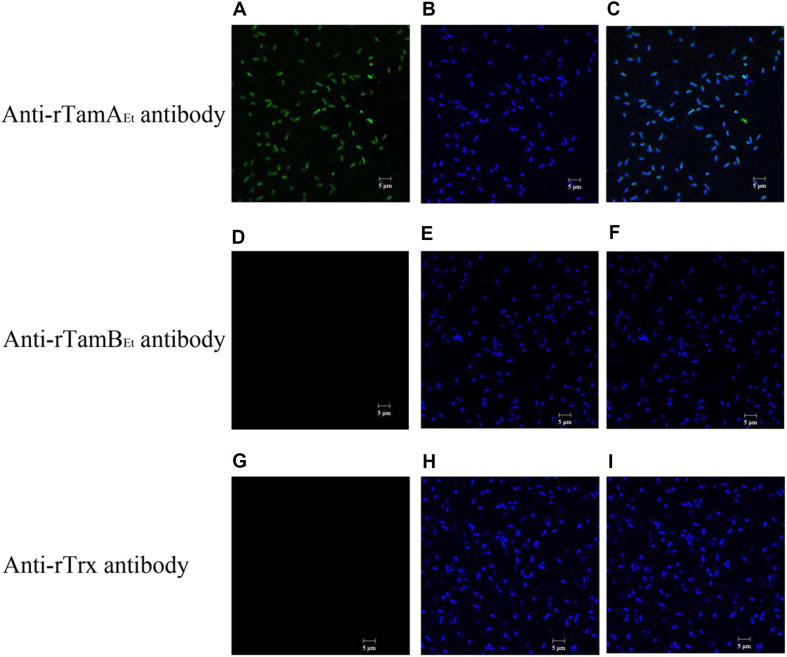
Detection of TamA_Et_ on bacterial surface. *Edwardsiella tarda* TX01 was incubated with anti-rTamA_Et_ antibody **(A,B)**, anti-rTamB_Et_ antibody **(D,E)**, or anti-rTrx antibody **(G,H)** and then treated with FITC-labeled secondary antibody and stained with DAPI. The cells were subjected to microscopy with green (detecting FITC label; **A,D,G**) or blue (detecting DAPI stain; **B,E,H**) fluorescence light. **(C)** A merged image of **(A,B)**; **(F)** a merged image of **(D,E)**; **(I)** a merged image of **(G,H)**.

Immunofluorescence microscopy showed that when *E. tarda* was treated with anti-rTamA_Et_ antibody, the antibody was detected on the cells, whereas no cell-associated antibody was detected in *E. tarda* treated with anti-rTamB_Et_ antibody or anti-rTrx antibody (Figure 1), suggesting that TamA_Et_ is surface-exposed in *E. tarda*.

### Growth and Survival of *tamA*_Et_ and *tamB*_E_ Mutants Under Different Conditions

#### Growth and Motility

**FIGURE 2:**
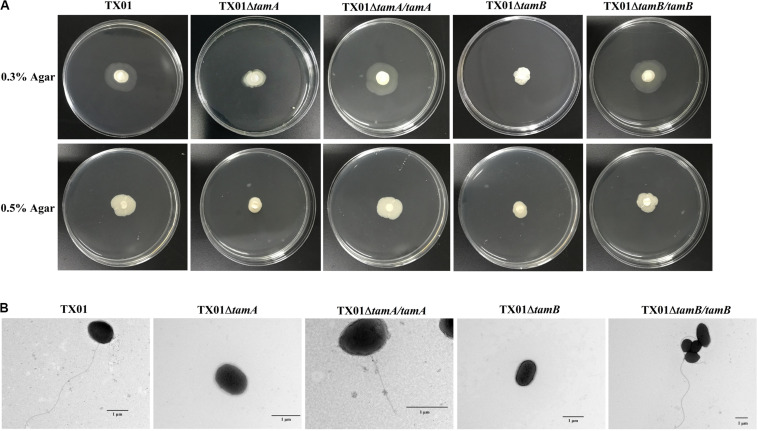
The motility and flagella of *Edwardsiella tarda* TX01 variants. **(A)** TX01, TX01Δ*tamA*, TX01Δ*tamB*, TX01Δ*tamA/tamA*, and TX01Δ*tamB/tamB* suspensions were spotted onto the center of LB plates containing 0.3 or 0.5% (w/v) agar. The plates were incubated at 28°C for 48 h before observation. **(B)** TX01, TX01Δ*tamA*, TX01Δ*tamB*, TX01Δ*tamA/tamA*, and TX01Δ*tamB/tamB* were cultured in LB medium and then examined with a transmission electron microscope.

Two isogenetic mutants of *E. tarda* TX01, i.e., TX01Δ*tamA* and TX01Δ*tamB*, were constructed, which bear markerless deletions of *tamA*_Et_ and *tamB*_Et_, respectively. The deletion of *tamB* in TX01Δ*tamB* was verified by Western blot, which detected no TamB production in TX01Δ*tamB* (Supplementary Figure S3). Growth analysis showed that when cultured in LB medium, TX01Δ*tamA* and TX01Δ*tamB*, displayed growth profiles similar to that of the parental strain TX01 (data not shown), which is consistent with previous observation in other bacterial species (Struve et al., 2003; Burall et al., 2004; Kelly et al., 2006; Selkrig et al., 2012). The swimming and swarming abilities of TX01Δ*tamA* and TX01Δ*tamB* were severely impaired in comparison to that of the wild type (Figure 2A and Table 3). In contrast, the swimming and swarming abilities of TX01Δ*tamA/tamA* and TX01Δ*tamB/tamB*, which have the *tamA*_Et_ and *tamB*_Et_ genes, respectively, introduced back to the mutant bacteria, were largely similar to that of the wild type (Figure 2A and Table 3). Electron microscopy showed that TX01, TX01Δ*tamA/tamA*, and TX01Δ*tamB/tamB*, but not TX01Δ*tamA* or TX01Δ*tamB*, possessed polar flagella (Figure 2B).

**TABLE 3:** The motility of *Edwardsiella tarda* TX01 variants.

	Diameter of swimming (mm)	Diameter of swarming (mm)
TX01	26.7 ± 1.5	21.3 ± 1.2
TX01Δ*tamA*	17 ± 1**	11.3 ± 1.2**
TX01Δ*tamA/tamA*	27.7 ± 1.5	20.7 ± 0.6
TX01Δ*tamB*	13.3 ± 2.3**	9.3 ± 1.5**
TX01Δ*tamB/tamB*	28.3 ± 0.6	19 ± 2

For the significance test, TX01 was used as a control for its variants. **P < 0.01.

#### Survival Under Acidic and Oxidizing Conditions

**FIGURE 3:**
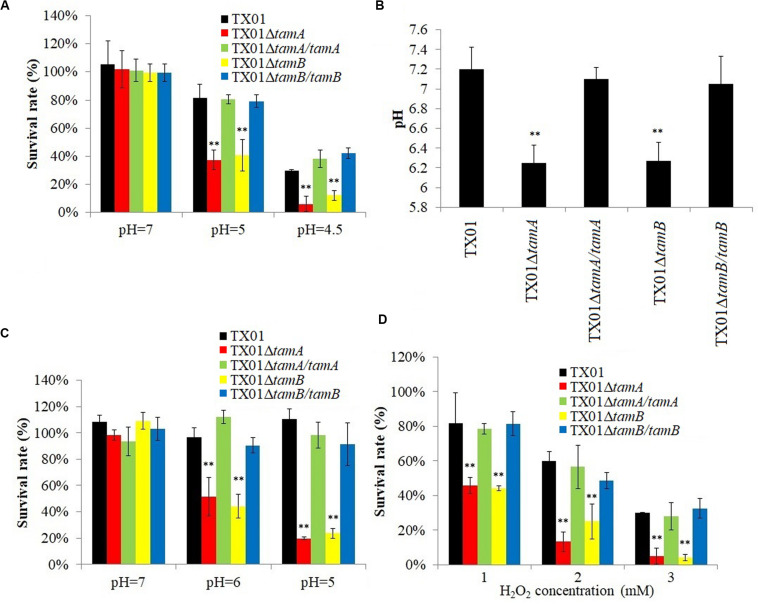
Effects of pH and H_2_O_2_ on the survival of *Edwardsiella tarda* variants. **(A)** TX01, TX01Δ*tamA*, TX01Δ*tamB*, TX01Δ*tamA/tamA*, and TX01Δ*tamB/tamB* were incubated in PBS of different pH for 2 h, and bacterial survival was determined. **(B)** TX01, TX01Δ*tamA*, TX01Δ*tamB*, TX01Δ*tamA/tamA*, and TX01Δ*tamB/tamB* were incubated in PBS of pH 5 for 2 h; after incubation, the pH of the cell lysate was determined. **(C)** TX01, TX01Δ*tamA*, TX01Δ*tamB*, TX01Δ*tamA/tamA*, and TX01Δ*tamB/tamB* were incubated in PBS of different pH for 2 h; after incubation, the cells were treated with lysozyme, and bacterial survival was determined. **(D)** TX01, TX01Δ*tamA*, TX01Δ*tamB*, TX01Δ*tamA/tamA*, and TX01Δ*tamB/tamB* were incubated with different concentrations of H_2_O_2_ for 1 h, and bacterial survival was determined. Data are the means of three independent assays and presented as means ± SEM. **P < 0.01.

When incubated in PBS buffer of pH 7, the survival rates of TX01Δ*tamA* and TX01Δ*tamB* were comparable to that of the wild type TX01. However, at pH 5 and pH 4.5, the survival rates of TX01Δ*tamA* and TX01Δ*tamB* were significantly lower than that of TX01 (Figure 3A). In contrast, the survival rates of TX01Δ*tamA/tamA* and TX01Δ*tamB/tamB* were largely similar to that of the wild type (Figure 3A). Furthermore, following incubation in the pH 5 buffer, the cell lysates of TX01Δ*tamA* and TX01Δ*tamB* exhibited pH values of 6.25 and 6.27, respectively, which were significantly lower than that of the cell lysates of the wild type (pH 7.2), TX01Δ*tamA/tamA* (pH 7.1) and TX01Δ*tamB/tamB* (pH 7.05) (Figure 3B). Pre-incubation in the pH 6 or pH 5 buffer, but not in the pH 7 buffer, significantly reduced the survival rates of TX01Δ*tamA* and TX01Δ*tamB* against lysozyme treatment (Figure 3C). In contrast, pre-incubation in low pH buffers had no significant effects on the survival of TX01Δ*tamA/tamA* or TX01Δ*tamB/tamB* against lysozyme treatment (Figure 3C). Similarly, the survival rates of TX01Δ*tamA* and TX01Δ*tamB*, but not TX01Δ*tamA/tamA* and TX01Δ*tamB/tamB*, in 1 mM, 2 mM, and 3 mM H_2_O_2_ were significantly reduced compared to that of the wild type (Figure 3D).

### Membrane Integrity of TX01Δ*tamA* and TX01Δ*tamB* Under Acidic Condition

**FIGURE 4:**
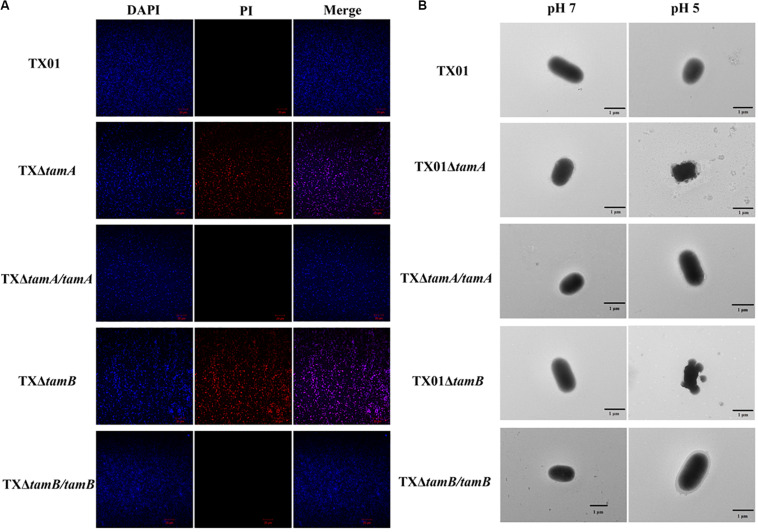
The structures of *Edwardsiella tarda* variants under acidic condition. **(A)** TX01, TX01Δ*tamA*, TX01Δ*tamB*, TX01Δ*tamA/tamA*, and TX01Δ*tamB/tamB* were incubated at pH 5 for 2 h and stained with DAPI and PI. The cells were subjected to microscopy with red or blue fluorescence light. **(B)** TX01, TX01Δ*tamA*, TX01Δ*tamB*, TX01Δ*tamA/tamA*, and TX01Δ*tamB/tamB* were incubated at pH 5 or pH 7 for 2 h and examined with a transmission electron microscope.

Fluorescence microscopy showed that when incubated in PBS buffer of pH 5, TX01Δ*tamA* and TX01Δ*tamB*, but not TX01, TX01Δ*tamA/tamA*, or TX01Δ*tamB/tamB*, were markedly labeled by PI (Figure 4A), which can only penetrate into dead or damaged cells. TEM showed that compared to pH 7, pH 5 induced severe damages to the cellular structures of TX01Δ*tamA* and TX01Δ*tamB*, with the bacteria cells exhibiting swelling and formation of bubble-like protrusion structures (Figure 4B). In contrast, no apparent change in the cellular structures of the wild type or the complemented strains was observed (Figure 4B).

### *In vitro* Infectivity of TX01Δ*tamA* and TX01Δ*tamB*

**FIGURE 5:**
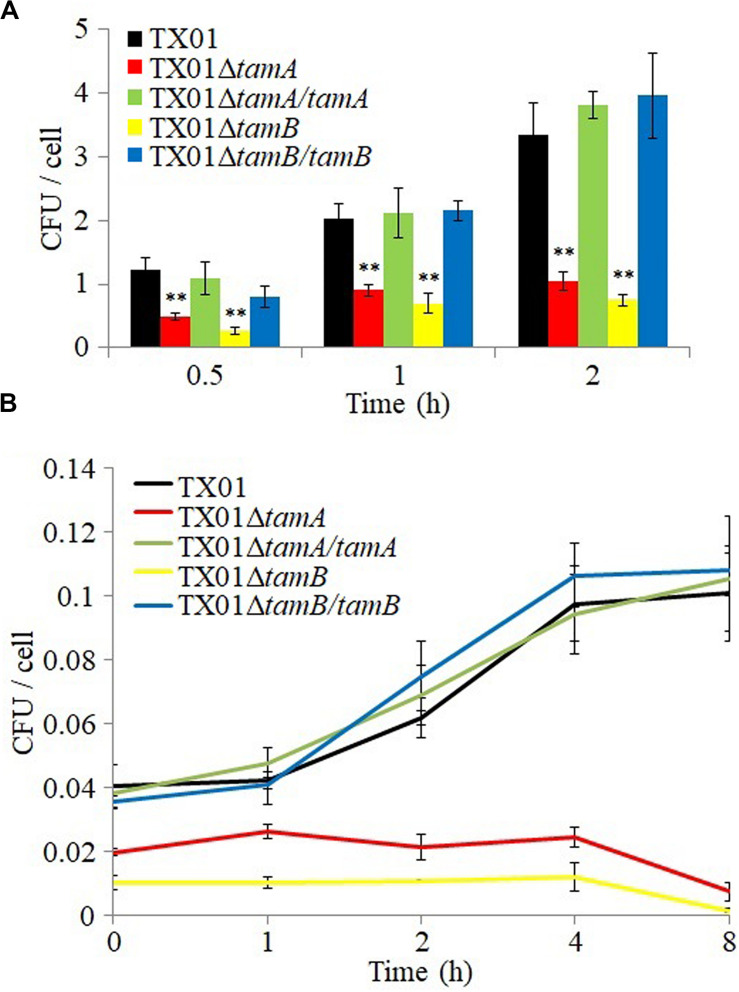
Cellular infectivity of *Edwardsiella tarda* variants. **(A)** Tongue sole peripheral blood leukocytes (PBL) were infected with TX01, TX01Δ*tamA*, TX01Δ*tamB*, TX01Δ*tamA/tamA*, or TX01Δ*tamB/tamB* for 0.5, 1, and 2 h, attached bacterial number was determined. **(B)** PBLs were infected as above for 1 h; the extracellular bacteria were killed with antibiotic, and the intracellular bacterial number was determined at different time points. Data are the means of three independent assays and presented as means ± SEM. **P < 0.01.

*In vitro* study showed that when tongue sole PBLs were infected with *E. tarda* mutants or wild type for 0.5, 1, and 2 h, the numbers of TX01Δ*tamA* and TX01Δ*tamB* recovered from the cells were significantly lower than that of the wild type TX01, whereas the numbers of recovered TX01Δ*tamA/tamA* and TX01Δ*tamB/tamB* were comparable to that of the wild type (Figure 5A). When the extracellular bacteria were removed by killing, the intracellular TX01, TX01Δ*tamA/tamA*, and TX01Δ*tamB/tamB* were found to continue to replicate and increase in number, whereas the intracellular TX01Δ*tamA* and TX01Δ*tamB* exhibited no detectable increase in number (Figure 5B).

### *In vivo* Infectivity and Lethality of TX01Δ*tamA* and TX01Δ*tamB*

**FIGURE 6:**
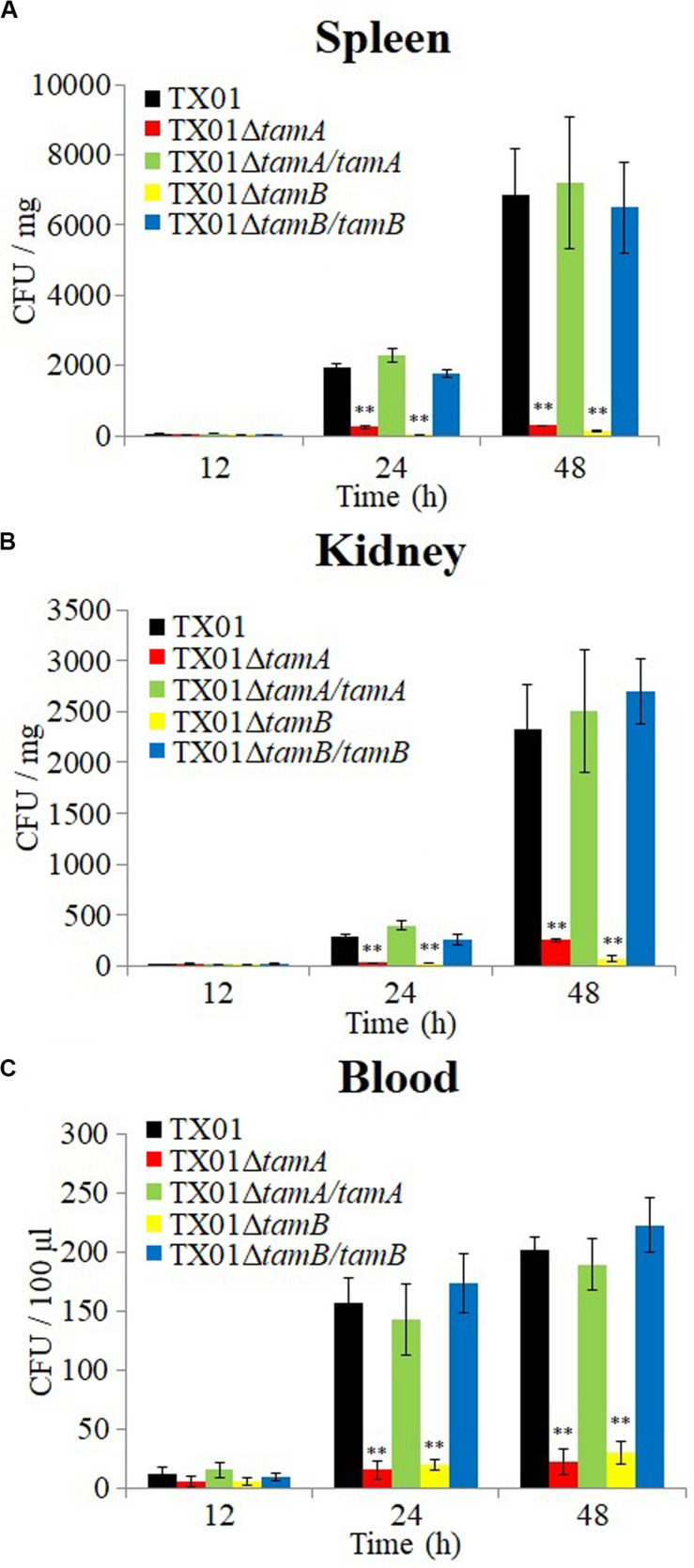
The tissue dissemination capacities of *Edwardsiella tarda* variants. Tongue sole were infected with TX01, TX01Δ*tamA*, TX01Δ*tamB*, TX01Δ*tamA/tamA*, or TX01Δ*tamB/tamB* for different hours, and bacterial recoveries from spleen **(A)**, kidney **(B)**, and blood **(C)** were determined. Data are the means of three independent assays and presented as means ± SEM. **P < 0.01.

**FIGURE 7:**
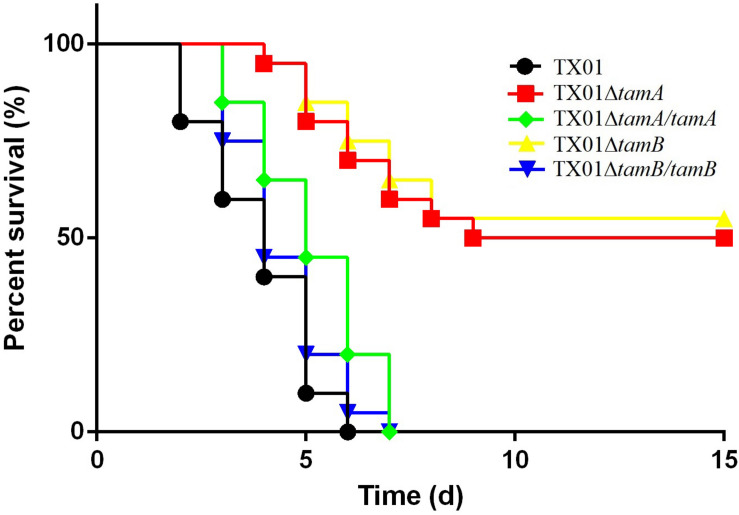
Survivals of tongue sole infected with *Edwardsiella tarda* variants. Tongue sole were infected with TX01, TX01Δ*tamA*, TX01Δ*tamB*, TX01Δ*tamA/tamA*, or TX01Δ*tamB/tamB*. The mortality and survival of the fish were monitored daily for 15 days. The experiment was performed three times, and the mean survival rates are shown.

*In vivo* study showed that when inoculated into tongue sole, TX01Δ*tamA* and TX01Δ*tamB* exhibited dramatically reduced bacterial disseminations in kidney, spleen, and blood in comparison to the wild type TX01, whereas the tissue dissemination capacities of TX01Δ*tamA/tamA* and TX01Δ*tamB/tamB* were similar to that of the wild type (Figure 6). Consistently, fish mortalities induced by TX01Δ*tamA* and TX01Δ*tamB* were significantly lower than that induced by TX01, TX01Δ*tamA/tamA*, or TX01Δ*tamB/tamB* (Figure 7).

## Discussion

In Gram-negative bacteria, the process of outer membrane assembly is dependent on the Omp85-family protein β-barrel assembly machinery (Hagan et al., 2011), and some membrane structures require a distinct subgroup of the Omp85 family protein, TamA (Selkrig et al., 2012; Gruss et al., 2013; Stubenrauch et al., 2016). TamA and BamA have similar domain structures, and both are Omp85-family proteins that function in parallel pathways for OMP assembly in bacteria (Selkrig et al., 2015). It has been postulated that in the evolution of some bacteria, duplicate copies of BamA form the origin of TamA (Heinz et al., 2015). In our study, we found that TamA_Et_ and TamB_Et_ possess conserved domains of TamA and TamB, respectively. TamA_Et_ has high levels of sequence identities with other bacterial OMP assembly factors but a low identity with the BamA of *E. tarda*, suggesting that the BAM and TAM complexes may have evolved to have different functions in *E. tarda.*

Gram-negative bacteria are separated from the external environment by the inner and outer membranes, which are set apart by the periplasm and the peptidoglycan layer (Albenne and Ieva, 2017). In Proteobacteria, TAM is composed of two membrane proteins, TamA and TamB. TamA is integrated in the outer membrane, while TamB has a signal-anchor sequence embedded in the inner membrane, and the interaction of these two subunits depends on TamB penetrating through the peptidoglycan layer (Selkrig et al., 2012; Shen et al., 2014). Consistently, we found that TamA_Et_, but not TamB_Et_, was exposed on the surface of *E. tarda*, suggesting that, like the TAM complex of other bacterial species (Selkrig et al., 2012), TamA_Et_ probably associates with the inner membrane protein TamB_Et_ and functions as a complex with the latter.

In uropathogenic *E. coli*, TAM catalyzes the assembly of the OMP FimD, which is essential for the ordered assembly of type 1 fimbriae (Munera et al., 2007; Stubenrauch et al., 2016). In *Proteus mirabilis*, transposon mutants of TamA and TamB show no defect in swarming activity (Burall et al., 2004). In our study, TX01Δ*tamA* and TX01Δ*tamB* exhibited no apparent flagella, implying a requirement of TamA_Et_ and TamB_Et_ in the biosynthesis/transport of *E. tarda* flagella. Consistently, TX01Δ*tamA* and TX01Δ*tamB* displayed very little swimming and swarming capacities. These observations indicated that *tamA*_Et_ and *tamB*_Et_ mutations affected flagella formation, resulting in impaired motility.

pH is known to regulate the expression of membrane proteins, such as porins, that are required for bacterial survival under stress conditions associated with low pH, oxidation, osmotic pressure, and high temperature (Begic and Worobec, 2006; Klimentova et al., 2019). Low pH has been shown to induce conformational changes in the extracellular loop reign of OmpG and influence the transmembrane pore formation (Korkmaz-Ozkan et al., 2010). At pH 5, OmpF increased fluoroquinolone antibiotic permeability and accumulation, resulting in increased antibiotic sensitivity (Cama et al., 2015). In *Mycobacterium bovis*, the transcription of the *ompA* gene was increased at pH 5.5 (Singh and Verma, 2008). In many pathogenic Gram-negative bacteria, the type III secretion system, which consists of the inner and OMP s and a needle, is also regulated by low pH (Markham et al., 2008). In our study, the survivals of TX01Δ*tamA* and TX01Δ*tamB* were significantly decreased under acidic and oxidative conditions, and the internal pH of these mutants were significantly affected by environmental pH, implying that the mutants were unable to maintain pH homeostasis inside the bacteria. Furthermore, low pH rendered TX01Δ*tamA* and TX01Δ*tamB* susceptible to lysozyme damage. Consistently, low pH severely damaged the cellular structures of TX01Δ*tamA* and TX01Δ*tamB*, which likely accounted for the observed lysozyme sensitivity under acidic conditions. These results suggested that TamA_Et_ and TamB_Et_ likely play an important role in the regulation of the proteins that form the normal membrane structure and determine the membrane integrity and permeability of *E. tarda*. It is also likely that TamA_Et_ and TamB_Et,_ by forming a complex, are directly involved in the formation of membrane integrity by constituting a membrane channel with selective permeability. *E. tarda* is an intracellular pathogen and can survive in host macrophages (Okuda et al., 2006; Liu et al., 2017). As a strategy of intracellular survival, *E. tarda* is able to detoxify the reactive oxygen species (ROS) generated by host phagocytes through the production of catalase and superoxide dismutases (Srinivasa Rao et al., 2003; Cheng et al., 2010). In our study, the survival rates of the TX01Δ*tamA* and TX01Δ*tamB* mutants after treatment with H_2_O_2_, which produces ROS, were significantly lower than that of the wild type, suggesting that the defective cellular structure caused by TamA_Et_ and TamB_Et_ mutation facilitated the H_2_O_2_-derived ROS to penetrate into the bacterial cells and kill the cells more effectively. This observation further supports the above conclusion that TamA_Et_ and TamB_Et_ are vital to the structural integrity of *E. tarda*.

The outer membrane of Gram-negative bacteria protects cells from external aggressions and mediates the secretion of virulence factors, and intact OMPs can promote host adhesion (Henderson and Nataro, 2001; Ranava et al., 2018). Efficient and correct assembly of integral OMPs requires the TAM complex (Heinz et al., 2015; Ranava et al., 2018). In *Citrobacter rodentium*, *Salmonella enterica* and *E. coli*, TAM mutation eliminated the virulence of the bacteria (Selkrig et al., 2012, 2014; Stubenrauch et al., 2016). In our study, we found that TX01Δ*tamA* and TX01Δ*tamB* exhibited significantly decreased ability to invade into and replicate in fish cells, and were significantly attenuated in the ability of tissue dissemination and inducing mortality in the host. These results indicated that TamA_Et_ and TamB_Et_ were required for optimal bacterial virulence. It is possible that mutation of *tamA*_Et_ and *tamB*_Et_ affects not only the membrane structure and permeability of the bacteria, but also the secretion of some virulence factors, which together lead to decreased survival in the stressing environment of the host.

## Conclusion

Our study demonstrated that TamA_Et_ and TamB_Et_ are required for motility, flagella development, stress survival, and infectivity of *E. tarda*. TamA_Et_ and TamB_Et_ exert a significant impact on membrane integrity, which affects both the physiology and the pathogenicity of the bacteria. These results add new insights into the function of bacterial TAM and the survival mechanism of *E. tarda*.

## Data Availability Statement

The datasets generated for this study can be found in the WP_012847252.1 of GenBank, WP_012847253.1 of GenBank.

## Ethics Statement

The animal study was reviewed and approved by the Ethics Committee of Institute of Oceanology, Chinese Academy of Sciences.

## Author Contributions

ML and LS conceived and designed the experiments. ML, BJ, and YS performed the experiments and analyzed the data. ML wrote the manuscript. LS edited the manuscript. All the authors contributed to the article and approved the submitted version.

## Conflict of Interest

The authors declare that the research was conducted in the absence of any commercial or financial relationships that could be construed as a potential conflict of interest.
