# Supplementary material for: The Translocation and Assembly Module (TAM) of Edwardsiella tarda Is Essential for Stress Resistance and Host Infection
Source: Front Microbiol. 2020 Jul 24;11:1743. doi: 10.3389/fmicb.2020.01743 (PMC7393178; doi:10.3389/fmicb.2020.01743)
Supplement: Supplementary file 1 [file Data_Sheet_1.docx]

**The** **translocation and assembly module (TAM) of *Edwardsiella tarda* is essential for stress resistance and host infection**

Mo-fei Li^1,2^, Bei-bei Jia^1,2,3^, Yuan-yuan Sun^1,2^, Li Sun^1,2^*

*^1^CAS Key Laboratory of Experimental Marine Biology, CAS Center for Ocean Mega-Science, Chinese Academy of Sciences, Institute of Oceanology, Qingdao, China*

*^2^Laboratory for Marine Biology and Biotechnology, Pilot National Laboratory for Marine Science and Technology (Qingdao), China*

***^3^****University of Chinese Academy of Sciences, Beijing, China*

*To whom correspondence should be addressed

Mailing address: Li Sun

Institute of Oceanology

Chinese Academy of Sciences

7 Nanhai Road

Qingdao 266071, China

Phone: 86-532-82898829

Email: lsun@qdio.ac.cn

Running title: The function of *E. tarda* TAM

**Supplemental data**

**Figure S1**．Domain structures of *Edwardsiella tarda* TamA (A) and TamB (B). TamA, outer membrane translocation and assembly module A; TamB, outer membrane translocation and assembly module B.

**
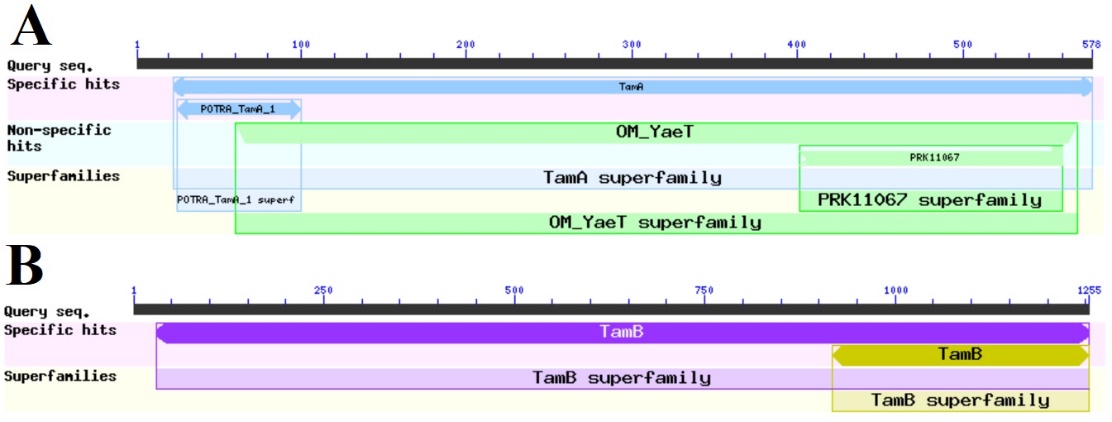
**

**Figure S2．**Pairwise sequence alignment of *Edwardsiella tarda* TamA and TamB homologues. Dots denote gaps introduced for maximum matching. Numbers in brackets indicate overall sequence identities between TamA_Et_ (A) and TamB_Et_ (B) homologues. The consensus residues are in red; the residues that are ≥75% identical among the aligned sequences are in black. (A) The GenBank accession numbers of the aligned sequences are as follows: *Edwardsiella ictaluri*, WP_015869878.1; *Edwardsiella hoshinae*, WP_024523503.1; *Hafnia alvei*, WP_072307805.1; *Hafnia paralvei*, WP_008814864.1; *Enterobacillus tribolii*, WP_115457381.1; *Yersinia rohdei*, WP_049616735.1; *Yersinia pseudotuberculosis*, WP_106440629.1; *Brenneria goodwinii*, WP_121589137.1. (B) The GenBank accession numbers of the aligned sequences are as follows: *Edwardsiella anguillarum*, WP_051905084.1; *Edwardsiella ictaluri*, WP_081167438.1; *Edwardsiella hoshinae*, WP_070245329.1; *Yersinia mollaretii*, WP_145558696.1; *Serratia marcescens*, WP_060388199.1; *Serratia grimesii*, HCK02992.1; *Klebsiella oxytoca*, WP_064290813.1; *Yersinia aleksiciae*, WP_145590373.1.


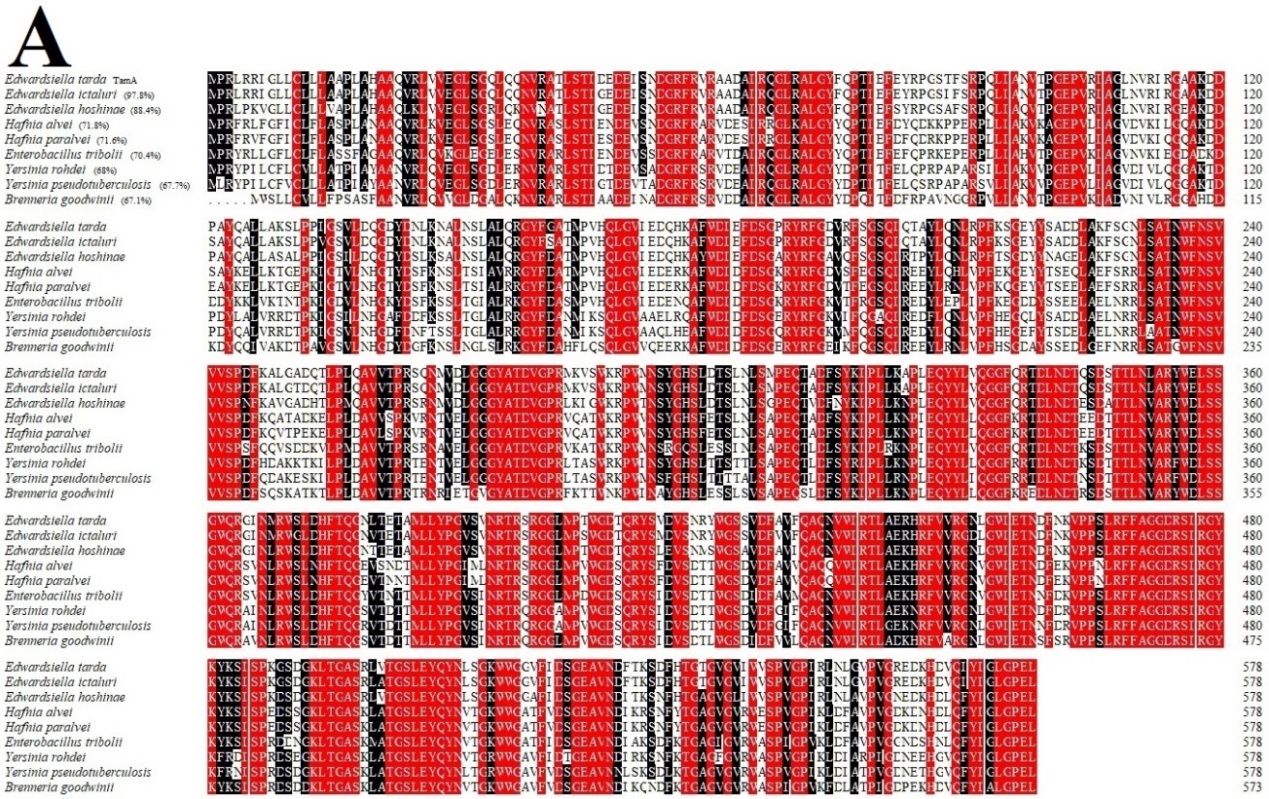


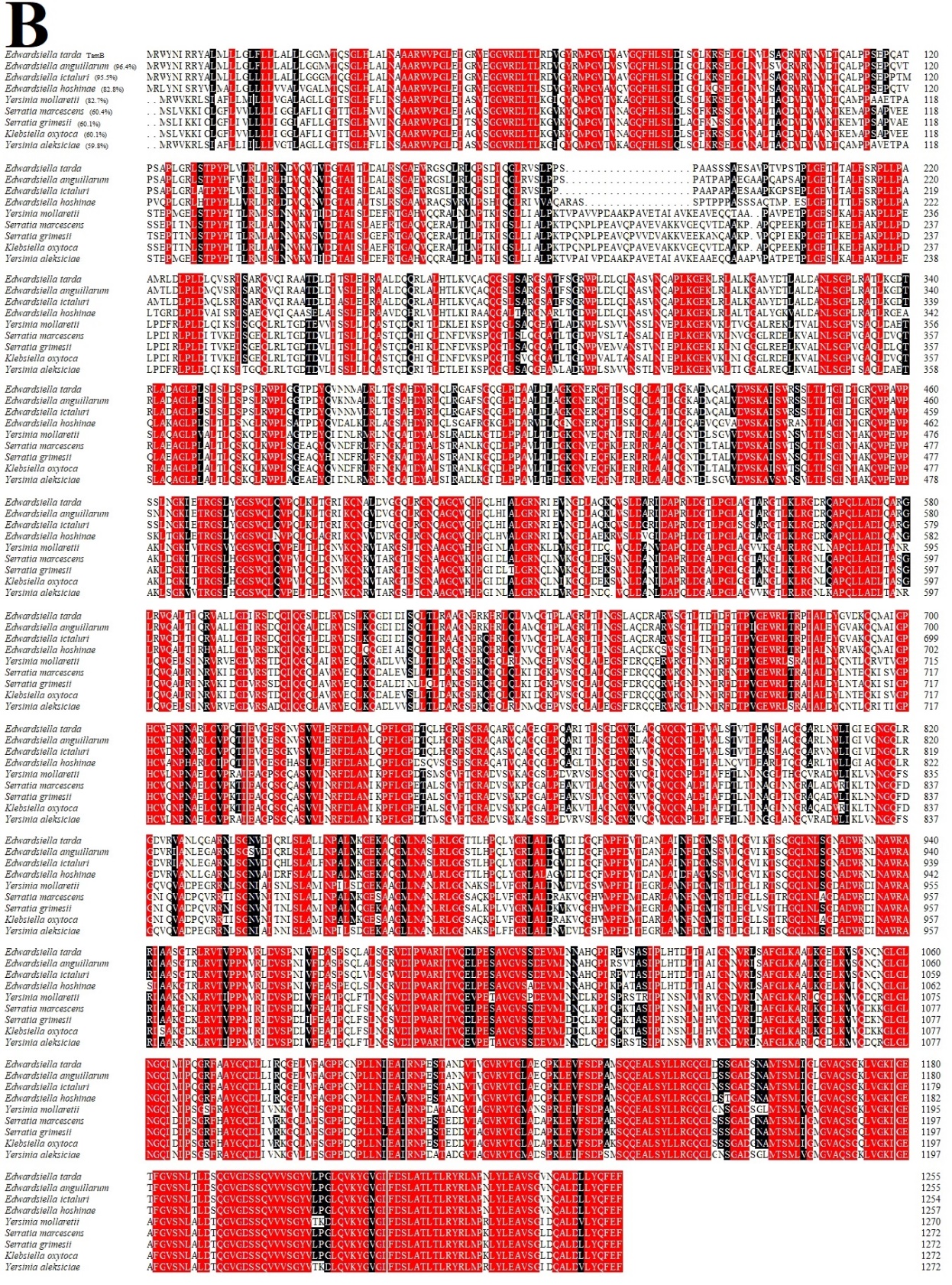


**Figure S3．**Immunoblot detection of TamB_Et_ in *Edwardsiella tarda* TX01 variants. The whole-cell proteins of TX01, TX01Δ*tamA*, TX01Δ*tamB*, TX01Δ*tamA/tamA*, or TX01Δ*tamB/tamB* were subjected to immunoblot analysis using Anti-rTamB_Et_ antibody. Anti-RNA polymerase beta antibody was used as an internal reference.

**
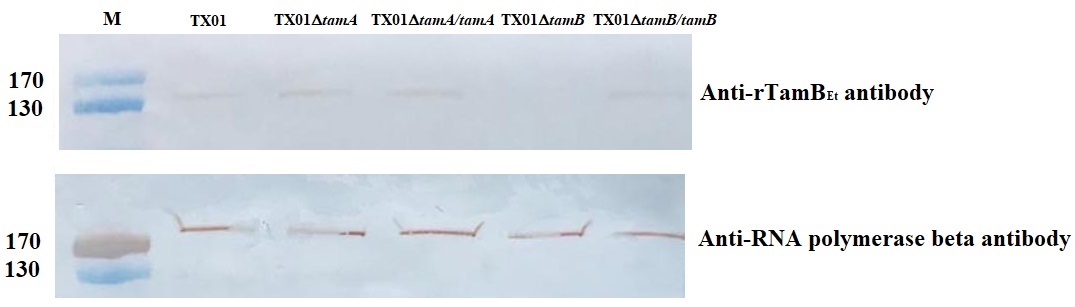
**
